# Manufacturing of animal products by the assembly of microfabricated tissues

**DOI:** 10.1042/EBC20200092

**Published:** 2021-08-06

**Authors:** Byeongwook Jo, Minghao Nie, Shoji Takeuchi

**Affiliations:** 1 Department of Mechano-Informatics, Graduate School of Information Science and Technology, The University of Tokyo, 7-3-1 Hongo, Bunkyo-ku, Tokyo 113-8656, Japan; 2 Institute of Industrial Science, The University of Tokyo, 4-6-1 Komaba, Meguro-ku, Tokyo 153-8505, Japan

**Keywords:** 3D bioprinting, cell layering, cultured meat, leather-like material, spinning

## Abstract

With the current rapidly growing global population, the animal product industry faces challenges which not only demand drastically increased amounts of animal products but also have to limit the emission of greenhouse gases and animal waste. These issues can be solved by the combination of microfabrication and tissue engineering techniques, which utilize the microtissue as a building component for larger tissue assembly to fabricate animal products. Various methods for the assembly of microtissue have been proposed such as spinning, cell layering, and 3D bioprinting to mimic the intricate morphology and function of the *in vivo* animal tissues. Some of the demonstrations on cultured meat and leather-like materials present promising outlooks on the emerging field of *in vitro* production of animal products.

## Introduction

Animal products are referred to as any product that is derived from animals. In the long history of human society, animal products have been utilized for a broad range of applications, from daily consumables such as meat, dairy products, leathers, to pharmaceutical products such as insulins for treating diabetes. With the currently rapidly growing global population [1], the animal product industry faces challenges which not only demand drastically increased amounts of animal products [2] but also have to limit the emission of greenhouse gases and animal waste [3]. Recently, transformative methods for animal product manufacture such as cultured animal products are drawing intense interest both in academia and in industry, and there have been some early successes such as extracting animal proteins through enzymatic methods for cosmetics and biological analysis. However, these methods cannot fabricate tissue-based animal products which are fabricated by culturing cells *in vitro*. Also, the fabricated tissues need to fulfill the morphological and functional characteristics of *in vivo* tissue are required; e.g., the animal meat tissue has highly aligned muscle fibers and well-distributed fat. For the fabrication of tissue-based animal products, recent technical advances in microfabrication and tissue engineering have led to techniques such as spinning [4–22], cell layering [23–28], and 3D bioprinting [29–64] to mimic intrinsic characteristics of *in vivo* animal tissue. In this review, we provide an overview of recent technological breakthroughs in the fabrication of tissue-based animal products such as cultured meat and leather-like materials.

## A brief history of animal products

Both the form of animal products and the way of obtaining animal products have evolved along with advances in human societies. Briefly, the evolution can be described with three stages marked by the three types of human societies: hunter-gathering society, agricultural society, and industrialized society. First, in hunter-gathering society, most of the animal products were not processed and consumed directly from the source such as meat, horn, and skin. Second, in an agricultural society, animal husbandry started developing which increased the number of domesticated animals and therefore increased the source of animal products in the form of not only meat and skin but also dairy products such as milk and eggs. In addition, processed animal products started to become popular such as fermented foods such as cheese and yogurt. Third, in industrialized society, the invention of dedicated machines has drastically increased the efficiency of animal husbandry and boosted the production of animal products. However, it is expected that the current animal production process will no longer be able to fulfill the needs of the rapidly growing population and the detailed reasons are as follows.

## Current challenges in animal products manufacturing

In 2030, the total global needs for animal products in developing countries are expected to increase by ∼50% [65]. Currently, more than three-quarters of the land used for agriculture is used for livestock. Assuming the production efficiency to be the same and considering the growth of the world population [1], it will be impossible to satisfy the global needs for animal products even all the land for agriculture is used for livestock. Also, current livestock farming systems have critical problems of greenhouse gas emissions, water consumption, a large volume of animal wastes [3]. Moreover, growing attention in animal ethics requires a new form of the production process for animal products [66]. To fulfill the current demands, instead of raising the livestock and slaughtering the livestock for production, researchers came up with the idea of producing the animal products within the lab facility, so-called cultured animal products. The cultured animal products procedures start from taking cells from an animal by biopsy, proliferating the number of cells, maturing the cells into tissue, then process the tissue into a target product [67,68]. However, tissue-based animal products, i.e., the animal products that mimic the functional and morphological characteristics of the *in vivo* animal tissues, have not yet been presented. The recent developments in the fabrication of microtissues and their assembly methods such as spinning, cell layering, and 3D bioprinting methods have shown the potential to fabricate scaled-up tissue with complexity in structure.

## Overview of the assembly methods of microtissues

The animal tissues such as skins, muscles, and organs have their unique function and structure. To fabricate animal tissues *in vitro*, scaffolding materials that can cause desirable cellular interactions to contribute to the formation of new functional tissues are required. Various material properties such as mechanical stiffness, chemical and surface properties are important for triggering desirable cell responses (e.g., differentiation of cells and secretion of ECMs) [69–72]. Moreover, proper microscale patterning of these properties is required to mimic the complex spatial–temporal distribution of factors during tissue development *in vivo*. To produce the microscale spatial–temporal patterning of these properties, the adaptation of microfabrication methods on tissue engineering has recently been proposed to fabricate microtissues which can be used as building blocks for large tissue assembly [73,74]. Here we introduce several assembly methods that can assemble into structures with high spatial resolution and multicompartmental characteristics.

### Spinning methods

Spinning refers to both the generation and handling of fiber-shaped materials applied in various manufacturing fields. The fiber-shaped materials are highly aligned structure with long length and flexibility which often function as the building blocks of not only clothes, construction cables but also animal body such as muscle and fur. Thus, constructing *in vitro* tissue with fiber-shaped materials presents the potentials to realize functional and morphological characteristics *in vivo*. To fabricate fiber-shaped materials using biomaterials, wet spinning and electrospinning are suitable methods among the various spinning methods.

#### Wet spinning

**Figure 1:**
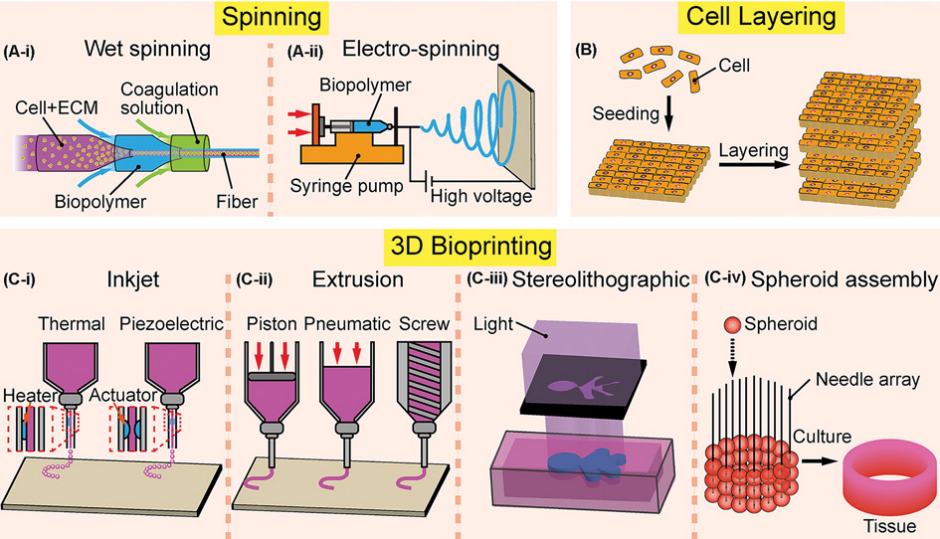
Various assembly methods of microtissues (**A**) Spinning methods produce fiber-shaped materials that can be used for biofabrication. (A-i) Wet spinning can fabricate fibers by cross-linking of the polymeric solution with coagulation medium through a microfluidic device in most cases. (A-ii) Electrospinning method can fabricate fibers into nanometer-scale through electrostatic repulsion and electric force applied to the droplet of polymer on the nozzle tip. (**B**) Cell layering is a fabrication process that can produce multilayered tissue through stacking, rolling, and deposition of the cell-laden biomaterials. (**C**) 3D bioprinting methods are categorized according to the different working principles. (C-i) Inkjet type bioprinters deposit droplets of bioink with precise control of the ejection volume within the nozzle which can be divided into two types. Thermal types utilize heaters to induce local vaporization that produces bubbles resulting in the generation of droplets. Piezoelectric types eject bioinks through a piezoelectric actuator equipped on the nozzle of the syringe. (C-ii) Extrusion types produce threads of bioinks through driving force such as piston, pneumatic pump, and screw. (C-iii) Stereolithographic types use light, mirrors, and lenses to polymerize the photoresponsive biomaterials which can be formed into the designed structure. (C-iv) Spheroid assembly type: spheroids are sucked by a printhead and dispensed on to desired substrates, such as needle array.

In wet spinning, a polymeric solution is extruded into a coagulation medium through a spinneret or microfluidic channels, then cross-linked into fibers (Figure 1(A-i)). The wet spun fibers can be divided into two groups which are non cell-laden fibers and cell-laden fibers. Non cell-laden fibers are used as scaffolds which require to be biocompatible, biodegradable with a structural condition for cell–cell and cell–material interactions [4]. Various biomaterials are used as polymeric solutions such as poly(lactide-co-glycolide) (PLGA) [6], chitosan [7,8], and alginate [9,10]. These wet spun fibers show porous structures with availability for cell adhesion and proliferation. Especially, highly oriented porous structure of fibers presents promising potentials for muscle tissue [4,11,12] and bone tissue [6,13] fabrications. In the case of cell-laden fibers, the mixture of cells and biopolymers are laden or encapsulated within the polymer through microfluidics device forming cell-laden fibers [14–16]. These cell-laden fibers possess a certain level of cellular morphologies and functions of tissue *in vivo* with high handleability that can be used as medical implantation. Also, since the wet spun cell-laden fibers are processed under gentle conditions with cell-friendly pH, osmolarity, temperature, and high cell viability can be achieved. The most distinct advantage of the wet spinning method is the capability of modulating the thickness, shape, and mechanical stiffness of the fibers through microfluidic channels. Owing to these advantages, assembly of larger scaffolds or tissues can be fabricated by manipulating wet spun fibers such as reeling [16,75–78], weaving [16,77,78], and bundling [4,79,80]. The components of the assembly can be adjustable with fibers containing different biomaterials and cell types. In addition, recent research presented scaled-up scaffold composed of fibrous gelatin which can be potentially useful for cultured meat production [4].

#### Electrospinning

Electrospinning is a fabrication process that can produce thin fibers down to nanometer scale from polymer solutions through electrostatic repulsion and electric field force. Electrospinning set up comprises a syringe with the polymer solution, collector plate, and high voltage supplier (Figure 1(A-ii)). While the polymer solution is extruded through the syringe, the high voltage applied to the syringe creates electrostatic repulsion of the extruded polymer that stretches the polymer cone-shaped droplet called Taylor cone [17]. Then, with the application of electric field forces, the polymer threads will be drawn from the tip of the Taylor cone and be accelerated towards the collector plate forming a fiber. The benefit of electrospinning is the capability of fabricating fibers down to a few hundreds of nanometers in diameter which can be used to induce cell behaviors such as controlling cell polarity [18–20]. Depending on the applied voltage and the density of polymer solutions, the diameter and the porosity of the electrospun fibers can be adjusted [81–83]. Also, using electrospinning, fibers containing cells [19–21] can be fabricated that are advantageous compared with other fabrication methods in terms of cell–cell interaction [22]. Through the accumulation of electrospun fibers, assembled scaffold or microtissue with high porosity can be achieved which is essential for the transportation of nutrients and oxygen [84]. Perspective applications for assembled electrospun fibers are skeletal muscle [85], bone [86,87] and blood vessels [88], but face some limitations on low mechanical properties and difficulties in controlling cell density of the fiber [22].

### Cell layering

A thin layer of cell-laden structures has been used for healing the wounds on the skin or internal organs. Cell layering is a method to assemble multiple thin layers of cell-laden structures to construct 3D-shaped tissue (Figure 1B). There are mainly three methods for cell layering: stacking of cell sheets [23], rolling of the cohesive tissue sheet [24–26], and *in situ* deposition of cell-laden biomaterials through a handheld printer [27,28]. First, the cell sheet is a single layer of cells harvested on a temperature-responsive polymer-coated culture dish. The temperature-responsive polymer is in a hydrophobic state at 37°C (the temperature used for cell culture) to stay attached to cell culture dishes, and changes to hydrophilic state at approx. 20°C to promote the detachment of cells from the culture dish. The cell sheets are then layered and cultured to form a multilayered tissue. The advantage of forming a cell sheet is easy and gentle detachment of cells keeping cell–cell and cell–ECM adhesion without damaging the cell membranes using enzymes such as trypsin. Second, fabrication of multilayered tissue through rolling is conducted by rolling a whole piece of thin tissue sheet around tubular support and culturing until tissue fusion. By rolling alternative types of tissue sheet on top of another, morphological structure of vascular constructs such as media and adventitia can be formed [24–26]. The rolling method has several advantages such as a simple fabrication process and the tissue thickness can be adjusted by the length of the to-be-rolled tissue sheet. Third, direct deposition of cell-laden biomaterials on a wound can be done through handheld apparatus [28]. The handheld apparatus is composed of a microfluidic dispenser in which the dispensing area can be adjusted by changing the width of the dispenser. The immediate cross-linking of fibrin-based bioink with thrombin solutions enables *in situ* fabrication of cell-laden biomaterials. The most distinct advantage of this method is a conformal deposition of the biomaterials even on the inclined surface [27].

### 3D bioprinting

The basic idea of 3D bioprinting is patterning the components of tissue such as cells and ECMs in a layer-by-layer additive manner. The materials used by the 3D bioprinting are called bioinks consisting of cells and biopolymers [29–34]. Depending on the printing methods, 3D bioprinting can be categorized into four main types/modalities: inkjet type, extrusion type, spheroid assembly type, and stereolithographic (SLA) type.

#### Inkjet types

One of the earliest forms of bioprinters is inkjet methods by depositing droplets of bioinks [35]. The bioinks are filled in a syringe or cartridge and formed into discrete droplets through a nozzle, then printed on to a substrate with precise control of localized pressure or force (Figure 1(C-i)). There are several types of inkjet bioprinters that utilize different printing methods such as the thermal method [36,38,39] and the piezoelectric method [40–43,89]. Thermal inkjet printers locally heat the nozzle through electric voltage, which triggers localized vaporization and induces bubbles that generate droplets of bioinks. Using the thermal inkjet method, high printing speed with a low cost can be achieved, but it is problematic since the cells are exposed under the condition of high temperature to undermine cell viability [37,44,45]. The piezoelectric inkjet printers apply acoustic waves through piezoelectric actuators equipped on the nozzle and eject droplets with controllable volume, but the applied acoustic waves (generally between 15 and 25 kHz) can induce damage to the cell membrane and lysis [38]. In summary, both the thermal and piezoelectric printers possess advantages of printing out at high speed with relatively low cost, while having disadvantages such as clogging of nozzle, inability to print bioinks with a viscosity higher than 12 mPa/s [46], and poor printability on the vertical direction which limits the fabricate a tissue similar to *in vivo* conditions.

#### Extrusion types

The extrusion bioprinters print out bioinks through a nozzle with the continuous driving force and fabricate desired structures on to the substrate with deposited threads of bioinks (Figure 1(C-ii)). Depending on the type of driving methods, extrusion bioprinters can be classified into three categories which are pneumatic, piston, and screw-driven bioprinters [47,90–92]. The extrusion bioprinters are capable of printing highly viscous bioinks with high cell density and are also feasible to fabricate a tissue with multiple cell types by simply adding extra printheads with different bioinks. Also, unlike inkjet bioprinters, the stacking of printed threads in the vertical direction is more feasible. Owing to these advantages, various applications are available such as fabricating perfusable channels by printing sacrificial materials [48–51] and forming a free-standing object without the use of supporting structures by extruding threads in the suspension baths [52,53]. However, extrusion types have drawbacks of clogging issues on a nozzle and require cautious optimization on the shear force during printing to avoid damages to the cells [54].

#### SLA types

The SLA bioprinters fabricate structure through polymerizing the photoresponsive bioinks. By exposing light using dynamically controlled mirrors and lenses, selective spots can be photocured (Figure 1(C-iii)). Various SLA bioprinters are presented such as direct laser writing (DLW) types [55,56] which draw traces of focused laser beam to polymerize the ink continuously, and digital light processing (DLP) types [57,58] which polymerize the whole layer at once through a device consisting of an array of digital mirrors. SLA printers can be easily adapted to print photocurable biomaterials with high resolution (5–50 µm) [58–60] which are suitable for fabricating refined structures like blood capillary and alveolar air sacs [61,62]. In addition, by designing bioinks that can polymerize under visible light [30], the SLA bioprinters can be also applied to print cell-laden bioinks. Moreover, SLA bioprinters mounted with microfluidic device provides multimaterial printability at a high spatial resolution [61,63]. The SLA bioprinters are advantageous due to high resolution and less concern of cell damages from shear stress. Comparing with the extrusion-based bioprinters which are compatible with a broad range of biomaterials, the SLA bioprinters are constrained to a few types of photocurable biomaterials such as poly(ethylene glycol) diacrylate (PEGDA) and gelatin methacrylate (GelMA). Also, since most of the SLA bioprinters utilize ultraviolet light for polymerization, concerns on damaging the cells need to be resolved. To further broaden the application of SLA bioprinters in tissue engineering, more types of photocurable biomaterials need to be developed [64].

#### Spheroid assembly types

Spheroid assembly bioprinters utilize spheroids as building blocks to fabricate centimeter-scale tissues. Before printing, spheroids are cultured in advance for the proliferation of cells. During printing, spheroids are sucked by a printhead and dispensed on to desired substrates, such as needle array (Figure 1(C-iv)). Since the closely contacting spheroids undergo a fusion process [93], the printed spheroids self-assemble into a single tissue. The most advantageous feature of the spheroid assembly bioprinter is that the fabricated tissues can achieve high cell density since the spheroids are formed using solely cells. Specifically, for transplantation applications, the tissues fabricated by the assembly of spheroids have the following advantages. First, patient cell-derived spheroids have the potential to relieve concerns of immunogenicity issues [94,95]. Second, not only various structures and sizes but also various types of cells can be modified according to clinical requirements. Using the spheroid assembly bioprinters, tubular cardiac constructs [94], and regeneration of diaphragm [96] are fabricated and show the potentials for applications in transplantation. In the future, further investigations on optimizing the cell sources and the maturation time are needed to improve the practical usability of the technique.

## Summary of the assembly methods

As summarized in Table 1, each assembly methods present advantages and disadvantages along with different resolutions and scalabilities. When fabricating microtissues *in vitro*, the selection of the assembly methods depends on the types and features of the target tissue. The following sections will present several case studies showcasing the detailed considerations on why and how each assembly method is chosen by and adapted to the needs of these specific cases.

**Table 1:** Outlines of various tissue assembly methods

Method	Advantages	Disadvantages	Resolution	Scalability
**Spinning**				
Wet spinning	Adjustable mechanical properties High cell viability	Complex fabrication process	7–250 μm [12–16]	∼1.5 mm [4]
Electrospinning	High porosity scaffold High resolution	Low mechanical stiffness Difficulty of controlling cell density	<1 μm [18,81–83]	∼1 mm [85]
**3D Bioprinting**				
Cell layering	Simple fabrication process Adjustable tissue thickness	Low planar resolution	Single-cell thickness [23]	Millimeter scale [101]
Inkjet type	High resolution (close to single cell)	Clogging of nozzle Poor printability on vertical direction Limited to low viscosity bioinks	30–100 μm [37–44]	∼400 μm [89]
Extrusion type	Printable with high viscosity bioinks Printable on vertical direction	Clogging of nozzle Damages on cells due to shear force	100–500 μm [47–54]	Centimeter scale [91,92]
Stereo-lithographic type	High spatial resolution Less concerns from shear force	Cell damages from UV light Limited types of bioinks	5–50 μm [58–63]	Centimeter scale [62]
Spheroid assembly type	Less concerns on immunogenicity High cell density	Low structural resolution Requires preparation of spheroids	Single spheroid size [93–96]	Centimeter scale [96]

## Case study

The fabrication of *in vitro* tissue has been developed mainly for biomedical applications such as biological analysis and regenerative medicine. The accumulated knowledge and know-how for constructing 3D-shaped cell-laden tissue-mimicking *in vivo* tissue morphology and function can also be used to create tissue-based animal products. However, the production of cultured animal products faces some technical issues to become commercially available such as scale-up fabrication and high production cost. To scale up the cell-laden tissue for animal products, structures such as blood vessels are required for supplying oxygen and nutrition which have not yet been achieved. Besides the scaling-up problem, reducing the production cost is another challenge. When the first demonstration of cultured meat by Mosa Meat was introduced, it cost more than $300,000 in 2013 [97]; 55–95% of the production cost originates from the culture media [98] which includes serums and other growth factors. Cost-effective methods to provide nutrition for the growth of cells through serum-free culture media are needed.

In this section, we will look into specific cases of animal product development and discuss how techniques on the assembly of microtissues are applied and how these technical issues are considered in real practice.

### Cultured meat

**Figure 2:**
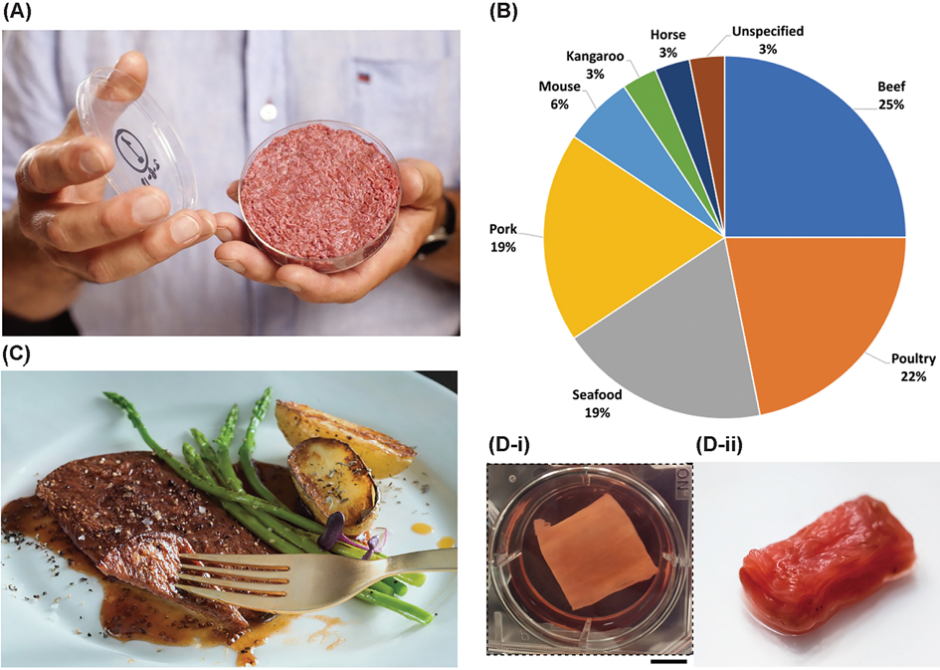
Recent progress on cultured meat (**A**) First cultured meat burger presented by Mosa Meat. (**B**) Pie graph showing the percentage of meat type aimed by cultured meat companies, the top shares are beef, poultry, seafood, and pork (data from 32 companies in 2020) (Reprinted with permission from [99], Copyright 2020 Elsevier.) (**C**) Thin-cut beef steak cultivated by Aleph Farms. (**D**-i) Muscle tissue in fibrous gelatin fabricated through spinning method (Reprinted with permission from [4], Copyright © 2019, MacQueen et al.) (**D**-ii) Millimeter-thick cultured steak using cell layering method (Reprinted with permission from [101]) Credit: Images reproduced with permission from Mosa Meat (A) and Aleph Farms (C).

The cultured meat aims to replicate animal meat under *in vitro* conditions. The process of cultured meat production starts from taking cells from a biopsy of the animal, then proliferating cells with nutrient supplements, and finally assembling the cells with biomaterials into 3D-shaped muscle tissue. Since the first demonstration of cultured meat hamburger in 2013 (Figure 2A), cultured meat has gathered great interest owing to its environmentally friendly production process compared with the meat from husbandry [3]. In 2020, 32 companies have been working on cultured meat including beef, pork, chicken, and shrimp [99] (Figure 2B), which can be used to cook dishes such as thin-cut steaks (Figure 2C) and meatballs. Despite some achievements on proliferating cells through bioreactors and adjusting the taste through food additives, the current culturing methods cannot produce meat that has uniformly aligned tissue structure in large scale (e.g. centimeter order). To mimic the densely packed structure of muscle which consists of fat and blood vessel, highly aligned structure of muscle in large scale is required. To overcome the current challenges, microtissue assembly methods are proposed to fabricate muscle tissue in large scale such as spinning [4], cell layering [100,101], 3D bioprinting [102,103] (Figure 2(D-i,ii)). Using spinning method, mass production of the fibrous gelatin was achieved that encourages adhesion of muscle cells to proliferate and align along the gelatin fiber [4]. Utilizing cell layering method, cell-laden hydrogel sheet with multilayered structure was fabricated fulfilling the functional characteristics of the muscle with contractile force [100,101] and similar breaking force (5.08 N) to that of the beef tenderloin (6.11 N) [101]. Through 3D bioprinting, muscle tissues with highly organized structure to guide cell alignments [105]. After the fabrication of muscle tissue, further maturation from 10 to 21 days were conducted to achieve functional and morphological characteristics of the muscle such as contractile force and striped patterns within the tissue (e.g. sarcomere) [100–103]. Since these presented examples are based on proof-of-concept, biomaterials such as fibrin, Matrigel, collagens, and serums are used to fabricate the *in-vitro* muscle tissue. However, these materials have food safety, cost, and ethical issues; since these materials are restricted to laboratory usages which are not approved by food administration, produced in small lot, and are all derived from animal tissues. To address these issues, there has been some emerging research directions such as plant-based edible biomaterials (e.g. soy protein [104,109–111]) and serum-free culture media. In addition, there have been some projects to probe the possibility of 3D printing cultured meat on the International Space Station [105]; the production speed in space is expected to be faster than on earth since the print can be done from all sides at once without the limitations of gravity.

### Cultured leather-like materials

**Figure 3:**
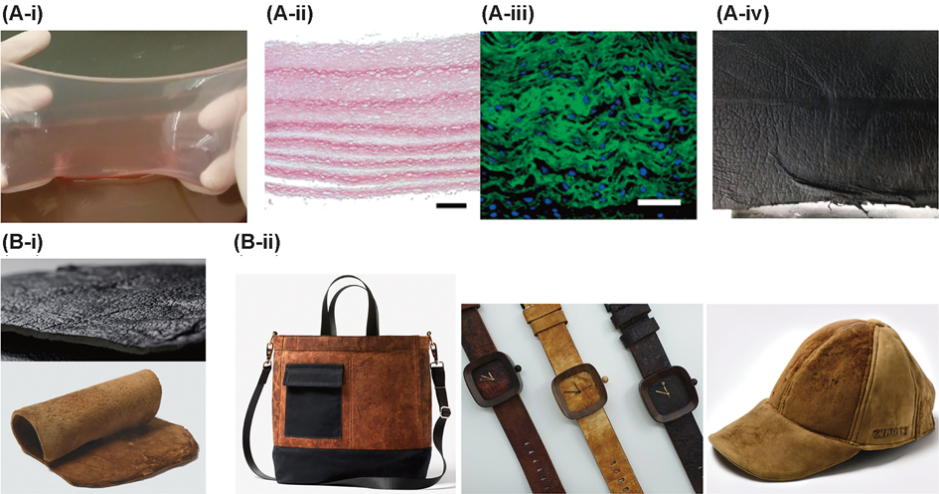
Prototypes of leather-like material products through cell layering and acellular agriculture (**A**-i) Cell-laden collagen sheet assembly with three layers of cell–ECM sheets. Histology of the 15-layer-thick tissue construct stained with (A-ii) PSR showing the collagen type I and (A-iii) immunofluorescent showing collagen type I (green), and cell nuclei (blue). Scale bar = 100 µm. (A-iv) Leather-like material after the process of tanning and dying showed physical properties that are comparable with natural leathers. (Reprinted with permission from [68], Copyright 2019 Elsevier.) (**B**-i) Leather like materials made from acellular agriculture which uses yeast cells or fungus to produce proteins. (B-ii) Fashion items such as bags, watch straps, and caps made from leather-like materials. Credit: Images reproduced with permission from Bolt Threads (B-i) top, (B-ii) bag. Mycotech (B-ii) watch strap. ZVNDER (B-i) bottom, and (B-ii) cap.

Leather products are processed from the animal skin composed of epidermis and dermis that are mainly made from proteins such as collagen and elastin. The essential techniques for leather-like materials originate from culturing the skin equivalent, which initially aimed at biological studies and medical applications [106–108,112–114] such as skin transplantation [109–111]. Using similar techniques for skin equivalent, cultured animal skin can be created to become the raw material for leather-like material production. By doing so, less animal brewing can be achieved not only to alleviate the growing concern on animal ethics but also to decrease the consumption of natural resources. The prototype of leather-like material was fabricated by first incorporating collagen-secreting fibroblasts into cell–ECM sheets and subsequently layering the cell–ECM sheets to form a multilayered tissue structure (Figure 3(A-i,iii)). After further maturation for the fusion of each cell–ECM sheet, the tanning process was conducted to modify the mechanical and chemical characteristics of the multilayered tissue structure into leather-like material (Figure 3(A-iv)). This leather-like material is yet early to be called leather but showed some physical properties such as tensile strength, elongation at break point and tear strength that are comparable with generally utilized upholstery leather (75 N, 30–80%, and 10 MPa, respectively) [68]. Going beyond the proof-of-concept into industrialized mass production, the cultured skin should reach certain thickness (at least 1 mm for upholstery) and resolve the problems such as lack of raw material supply; biomaterials for producing skin tissues such as collagen were difficult to mass-produce. Recently, researchers came up with the methods to mass-produce the biomaterials called acellular agriculture, which utilizes genetically modified yeast cells or fungus to mass-produce biomaterials for leather-like materials [112–114] (Figure 3(B-i)). As shown in Figure 3(B-ii), companies working on mass production of protein from acellular agriculture made leather-like materials that can be used as fashion items such as bags, watch straps, and caps. In addition, through acellular agriculture, biomaterials such as collagens can be grown within few weeks and the produced biomaterials possess the mechanical characteristics that are comparable with natural leather.

Although fabrication of leather-like materials still faces the long journey to fully mimic the characteristics of the natural leather, environment-friendly production process and flexibility of the shape during the fabrication present a positive outlook to be considered as another option for the apparel industry.

Finally, further efforts on acellular agriculture and fabrication of microtissue into cultured animal skin will not only accelerate the timeline for mass production but also lead to leather-like products that have not been presented from conventional leather products from animal skin.

## Summary

- With the current rapidly growing global population, the animal product industry faces challenges such as the drastically increased demands on the amounts of animal products, reduction in the emission of greenhouse gases, and animal waste.
- The combination of microfabrication and tissue engineering techniques has led to the fabrication of microtissue which can be used as building blocks for large tissue assembly.
- Producing animal products through the assembly of microtissues is gathering attention due to alternative methods to overcome the limitations of the current production process.
- Recent attempts on manufacturing animal products *in vitro* introduced some demonstrations of cultured meat and leather-like materials which have shown the ability to mimic the intricate morphology and function of *in vivo* animal tissue.

## Open Access

Open access for this article was enabled by the participation of University of Tokyo in an all-inclusive *Read & Publish* pilot with Portland Press and the Biochemical Society under a transformative agreement with USACO.

## Abbreviations

ECM: extracellular matrix
SLA: stereolithographic
